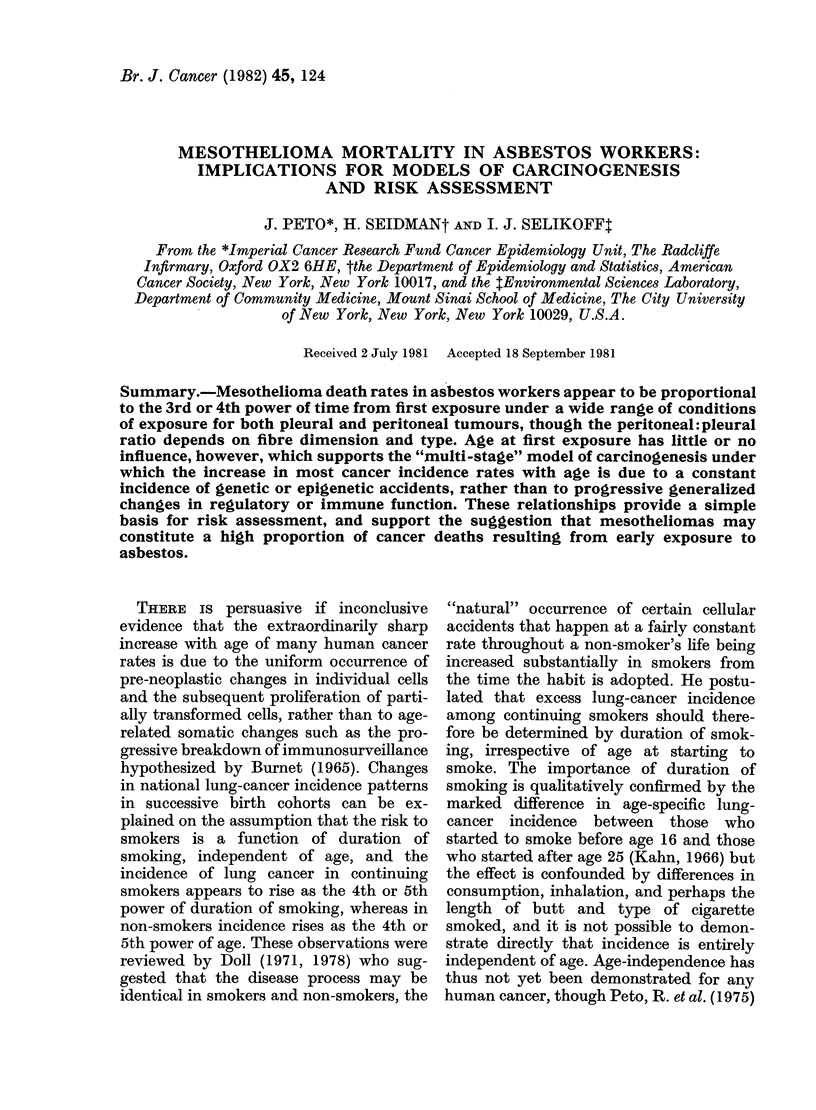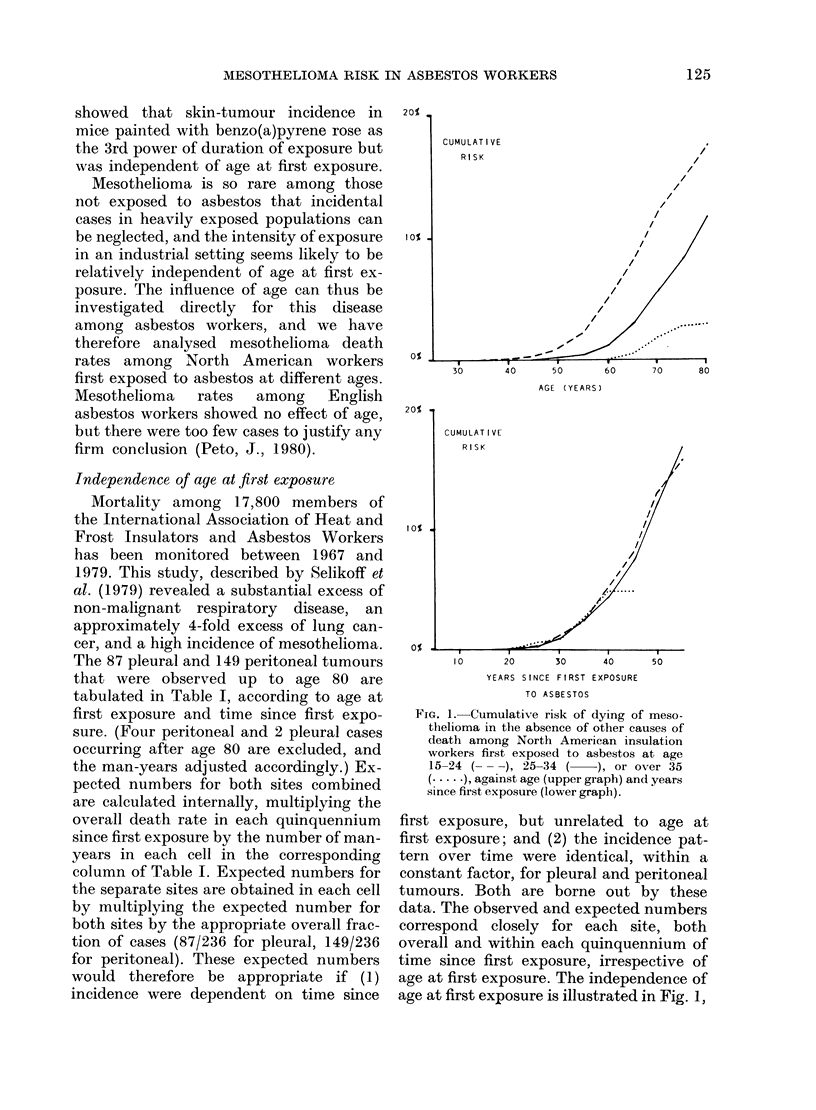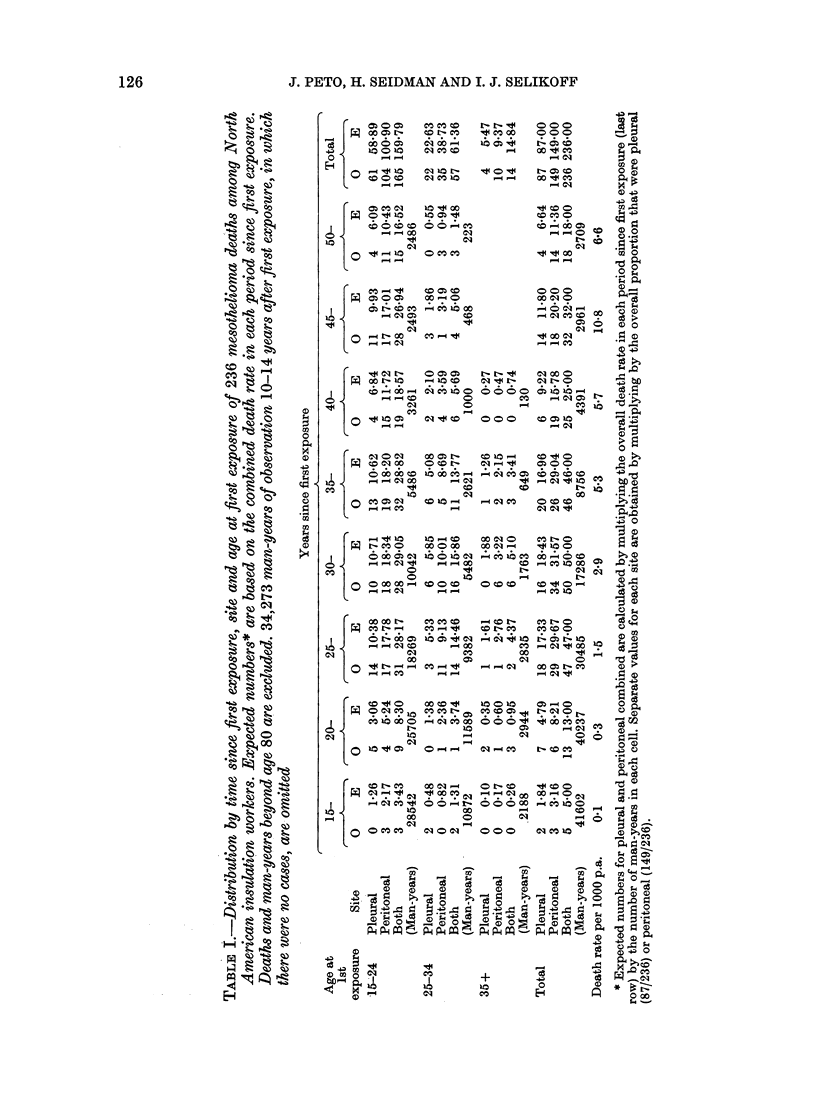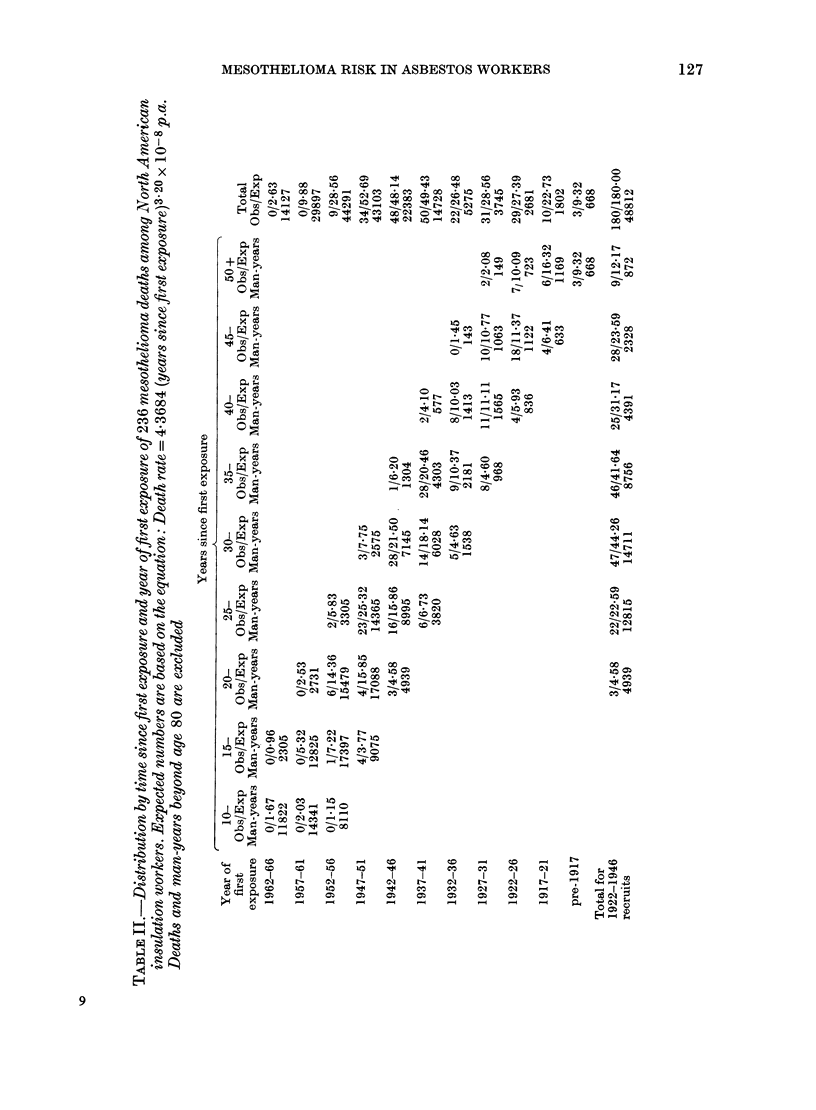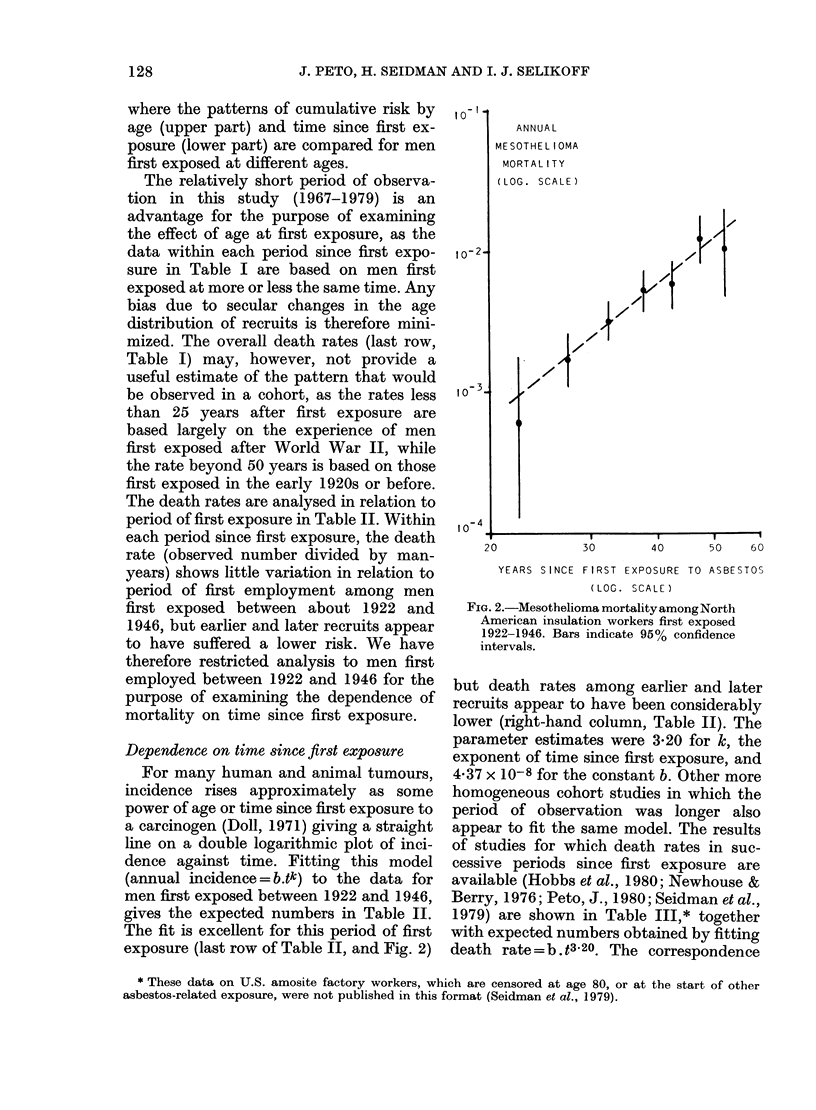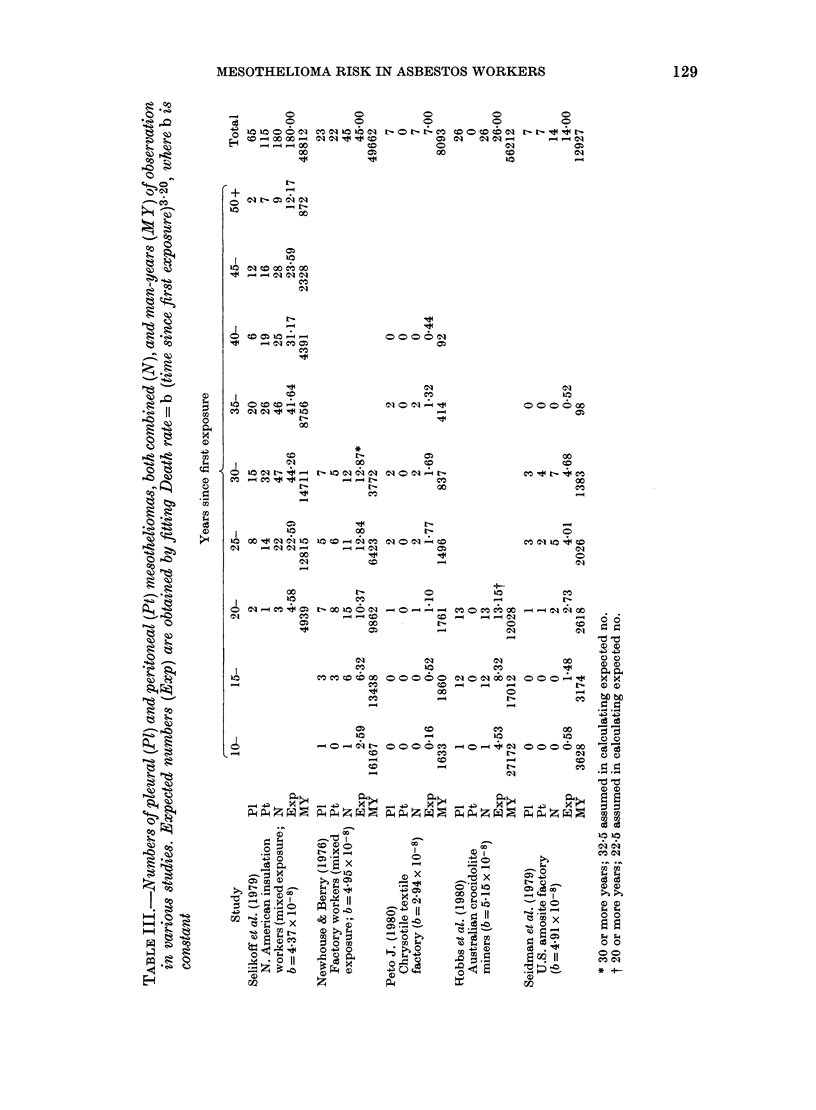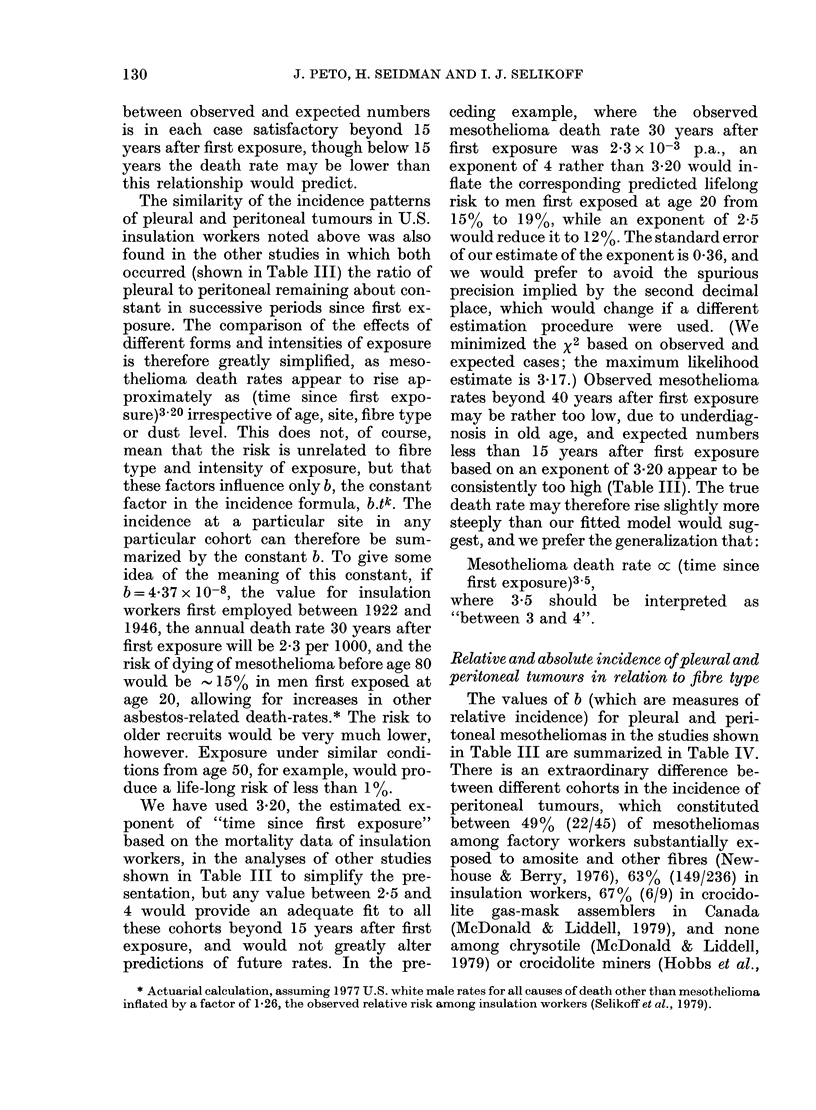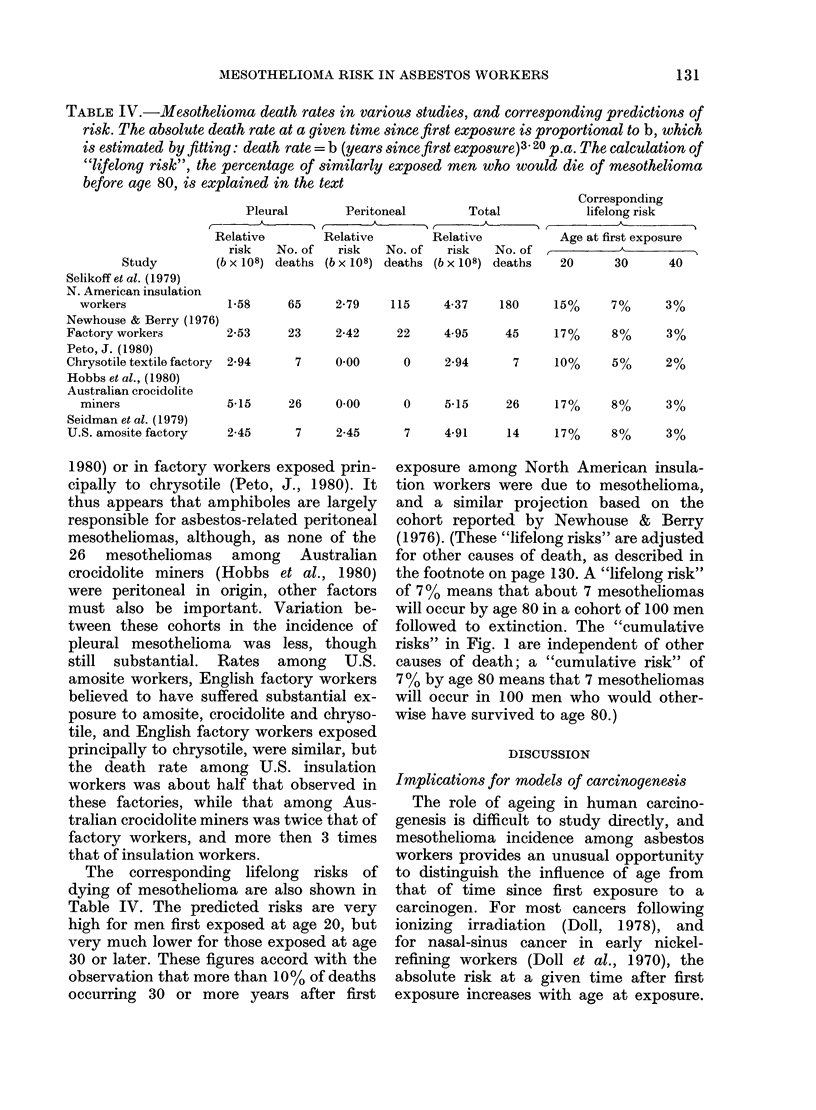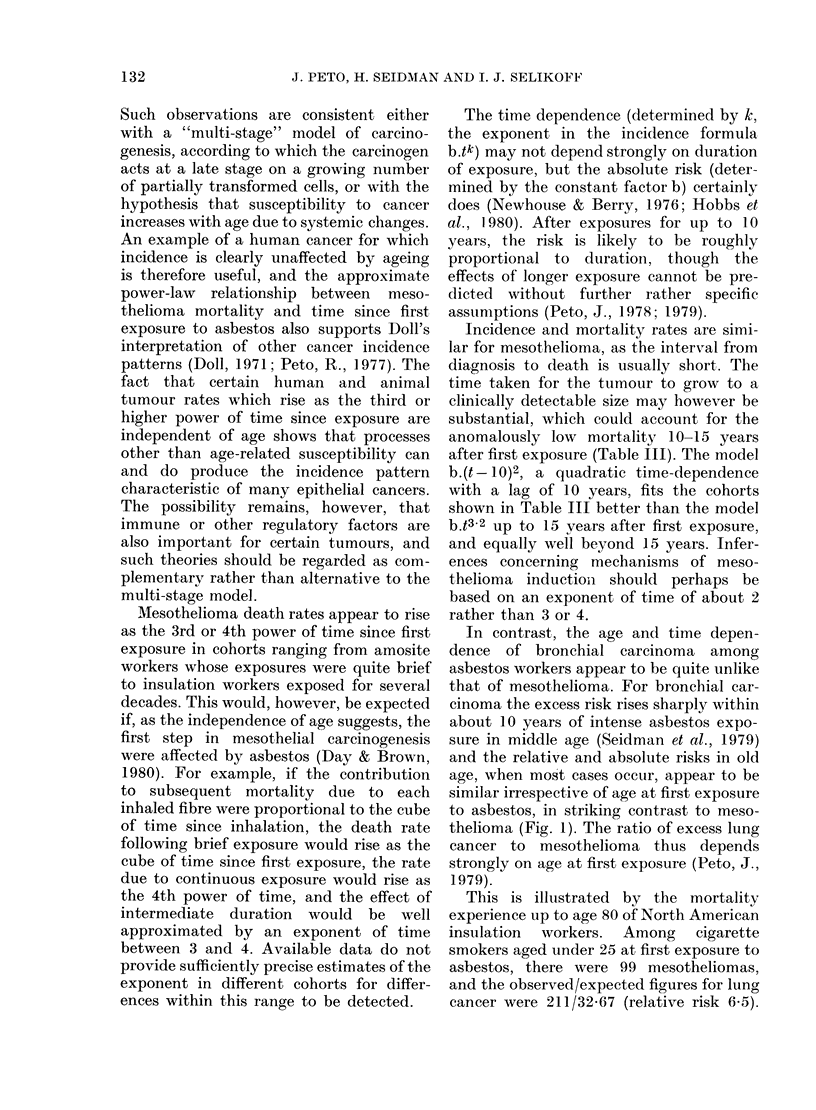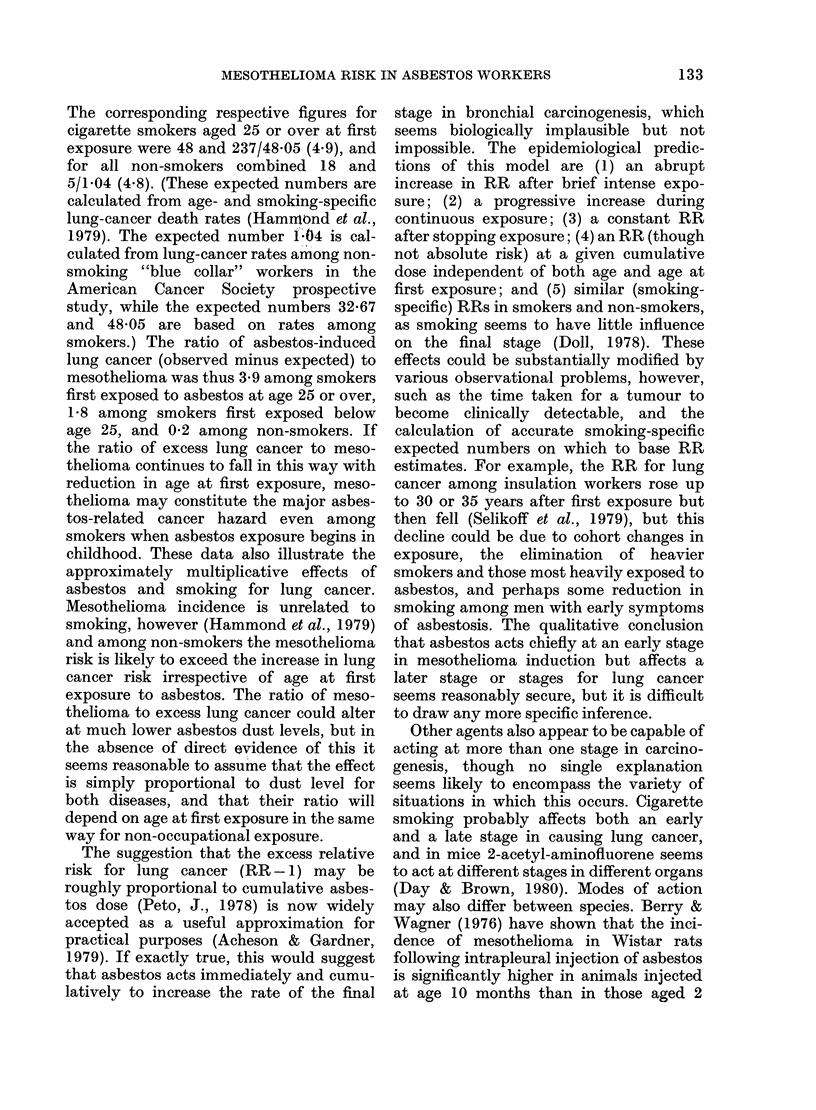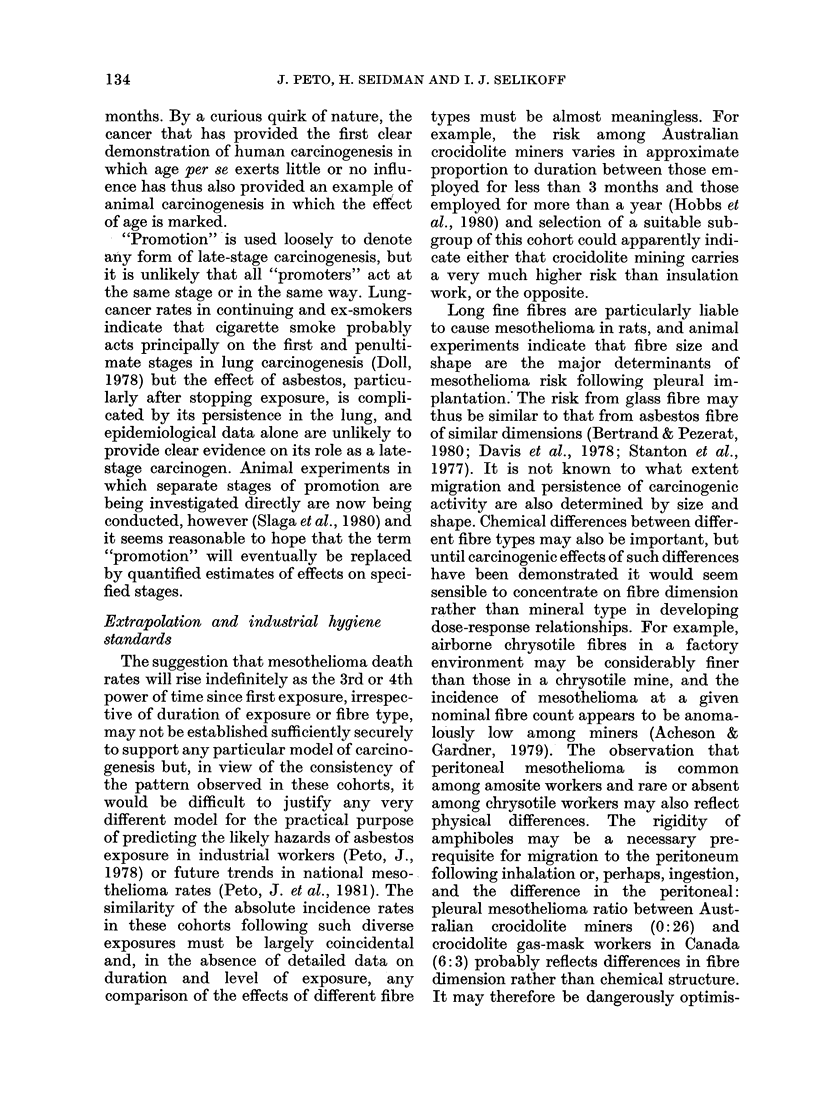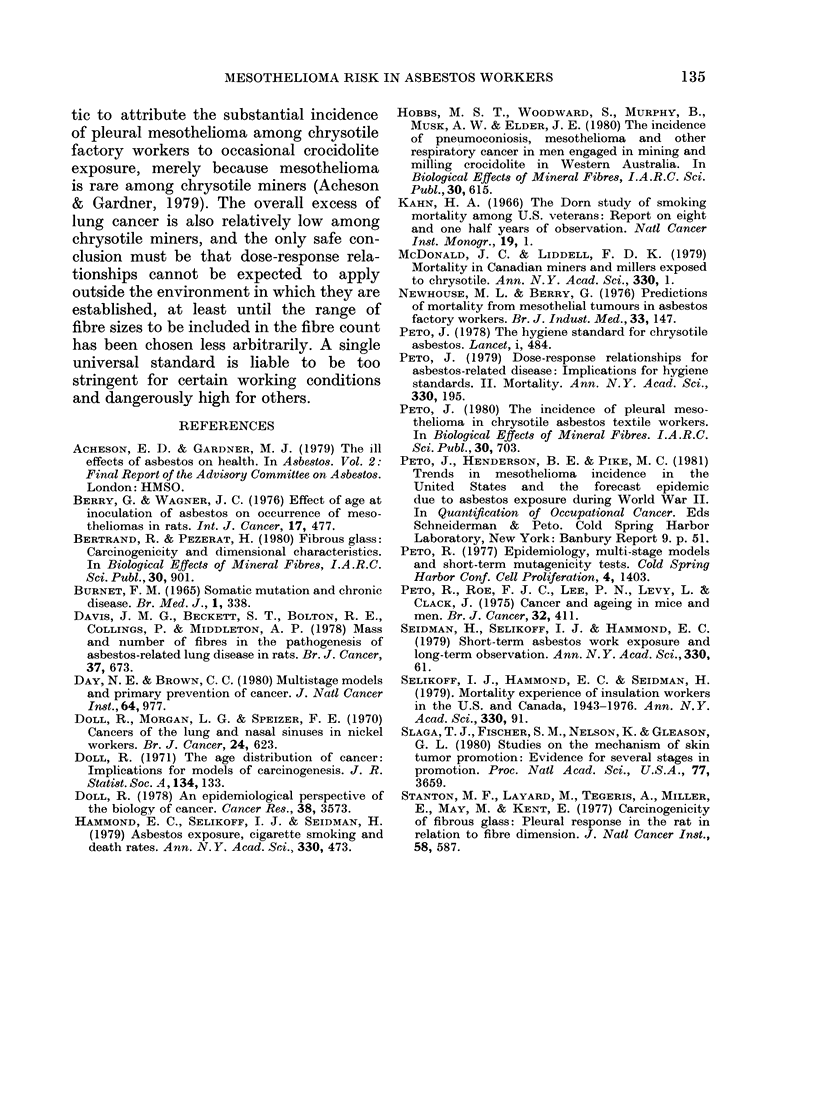# Mesothelioma mortality in asbestos workers: implications for models of carcinogenesis and risk assessment.

**DOI:** 10.1038/bjc.1982.15

**Published:** 1982-01

**Authors:** J. Peto, H. Seidman, I. J. Selikoff

## Abstract

Mesothelioma death rates in asbestos workers appear to be proportional to the 3rd or 4th power of time from first exposure under a wide range of conditions of exposure for both pleural and peritoneal tumours, though the peritoneal:pleural ratio depends on fibre dimension and type. Age at first exposure has little or no influence, however, which supports the "multi-stage" model of carcinogenesis under which the increase in most cancer incidence rates with age is due to a constant incidence of genetic or epigenetic accidents, rather than to progressive generalized changes in regulatory or immune function. These relationships provide a simple basis for risk assessment, and support the suggestion that mesotheliomas may constitute a high proportion of cancer deaths resulting from early exposure to asbestos.


*Br. J. Cancer* (1982) **45**, 124

### MESOTHELIOMA MORTALITY IN ASBESTOS WORKERS: IMPLICATIONS FOR MODELS OF CARCINOGENESIS AND RISK ASSESSMENT

J. PETO*, H. SEIDMAN† AND I. J. SELIKOFF‡

*From the *Imperial Cancer Research Fund Cancer Epidemiology Unit, The Radcliffe Infirmary, Oxford OX2 6HE, †the Department of Epidemiology and Statistics, American Cancer Society, New York, New York* 10017, *and the ‡Environmental Sciences Laboratory, Department of Community Medicine, Mount Sinai School of Medicine, The City University of New York, New York, New York* 10029, *U.S.A.*

Received 2 July 1981 Accepted 18 September 1981

**Summary.—Mesothelioma death rates in asbestos workers appear to be proportional to the 3rd or 4th power of time from first exposure under a wide range of conditions of exposure for both pleural and peritoneal tumours, though the peritoneal:pleural ratio depends on fibre dimension and type. Age at first exposure has little or no influence, however, which supports the "multi-stage" model of carcinogenesis under which the increase in most cancer incidence rates with age is due to a constant incidence of genetic or epigenetic accidents, rather than to progressive generalized changes in regulatory or immune function. These relationships provide a simple basis for risk assessment, and support the suggestion that mesotheliomas may constitute a high proportion of cancer deaths resulting from early exposure to asbestos.**

THERE IS persuasive if inconclusive evidence that the extraordinarily sharp increase with age of many human cancer rates is due to the uniform occurrence of pre-neoplastic changes in individual cells and the subsequent proliferation of partially transformed cells, rather than to age-related somatic changes such as the progressive breakdown of immunosurveillance hypothesized by Burnet (1965). Changes in national lung-cancer incidence patterns in successive birth cohorts can be explained on the assumption that the risk to smokers is a function of duration of smoking, independent of age, and the incidence of lung cancer in continuing smokers appears to rise as the 4th or 5th power of duration of smoking, whereas in non-smokers incidence rises as the 4th or 5th power of age. These observations were reviewed by Doll (1971, 1978) who suggested that the disease process may be identical in smokers and non-smokers, the "natural" occurrence of certain cellular accidents that happen at a fairly constant rate throughout a non-smoker's life being increased substantially in smokers from the time the habit is adopted. He postulated that excess lung-cancer incidence among continuing smokers should therefore be determined by duration of smoking, irrespective of age at starting to smoke. The importance of duration of smoking is qualitatively confirmed by the marked difference in age-specific lung-cancer incidence between those who started to smoke before age 16 and those who started after age 25 (Kahn, 1966) but the effect is confounded by differences in consumption, inhalation, and perhaps the length of butt and type of cigarette smoked, and it is not possible to demonstrate directly that incidence is entirely independent of age. Age-independence has thus not yet been demonstrated for any human cancer, though Peto, R. *et al.* (1975)

showed that skin-tumour incidence in mice painted with benzo(a)pyrene rose as the 3rd power of duration of exposure but was independent of age at first exposure.

Mesothelioma is so rare among those not exposed to asbestos that incidental cases in heavily exposed populations can be neglected, and the intensity of exposure in an industrial setting seems likely to be relatively independent of age at first exposure. The influence of age can thus be investigated directly for this disease among asbestos workers, and we have therefore analysed mesothelioma death rates among North American workers first exposed to asbestos at different ages. Mesothelioma rates among English asbestos workers showed no effect of age, but there were too few cases to justify any firm conclusion (Peto, J., 1980).

*Independence of age at first exposure*

Mortality among 17,800 members of the International Association of Heat and Frost Insulators and Asbestos Workers has been monitored between 1967 and 1979. This study, described by Selikoff *et al.* (1979) revealed a substantial excess of non-malignant respiratory disease, an approximately 4-fold excess of lung cancer, and a high incidence of mesothelioma. The 87 pleural and 149 peritoneal tumours that were observed up to age 80 are tabulated in Table I, according to age at first exposure and time since first exposure. (Four peritoneal and 2 pleural cases occurring after age 80 are excluded, and the man-years adjusted accordingly.) Expected numbers for both sites combined are calculated internally, multiplying the overall death rate in each quinquennium since first exposure by the number of man-years in each cell in the corresponding column of Table I. Expected numbers for the separate sites are obtained in each cell by multiplying the expected number for both sites by the appropriate overall fraction of cases (87/236 for pleural, 149/236 for peritoneal). These expected numbers would therefore be appropriate if (1) incidence were dependent on time since first exposure, but unrelated to age at first exposure; and (2) the incidence pattern over time were identical, within a constant factor, for pleural and peritoneal tumours. Both are borne out by these data. The observed and expected numbers correspond closely for each site, both overall and within each quinquennium of time since first exposure, irrespective of age at first exposure. The independence of age at first exposure is illustrated in Fig. 1,

FIG. 1.—Cumulative risk of dying of mesothelioma in the absence of other causes of death among North American insulation workers first exposed to asbestos at age 15–24 (– – –), 25–34 (——), or over 35 (· · · · ·), against age (upper graph) and years since first exposure (lower graph).

where the patterns of cumulative risk by age (upper part) and time since first exposure (lower part) are compared for men first exposed at different ages.

The relatively short period of observation in this study (1967–1979) is an advantage for the purpose of examining the effect of age at first exposure, as the data within each period since first exposure in Table I are based on men first exposed at more or less the same time. Any bias due to secular changes in the age distribution of recruits is therefore minimized. The overall death rates (last row, Table I) may, however, not provide a useful estimate of the pattern that would be observed in a cohort, as the rates less than 25 years after first exposure are based largely on the experience of men first exposed after World War II, while the rate beyond 50 years is based on those first exposed in the early 1920s or before. The death rates are analysed in relation to period of first exposure in Table II. Within each period since first exposure, the death rate (observed number divided by man-years) shows little variation in relation to period of first employment among men first exposed between about 1922 and 1946, but earlier and later recruits appear to have suffered a lower risk. We have therefore restricted analysis to men first employed between 1922 and 1946 for the purpose of examining the dependence of mortality on time since first exposure.

*Dependence on time since first exposure*

For many human and animal tumours, incidence rises approximately as some power of age or time since first exposure to a carcinogen (Doll, 1971) giving a straight line on a double logarithmic plot of incidence against time. Fitting this model (annual incidence $= b.t^k$) to the data for men first exposed between 1922 and 1946, gives the expected numbers in Table II. The fit is excellent for this period of first exposure (last row of Table II, and Fig. 2)

FIG. 2.—Mesothelioma mortality among North American insulation workers first exposed 1922–1946. Bars indicate 95% confidence intervals.

but death rates among earlier and later recruits appear to have been considerably lower (right-hand column, Table II). The parameter estimates were 3·20 for $k$, the exponent of time since first exposure, and $4{\cdot}37 \times 10^{-8}$ for the constant $b$. Other more homogeneous cohort studies in which the period of observation was longer also appear to fit the same model. The results of studies for which death rates in successive periods since first exposure are available (Hobbs *et al.*, 1980; Newhouse & Berry, 1976; Peto, J., 1980; Seidman *et al.*, 1979) are shown in Table III,* together with expected numbers obtained by fitting death rate $= \text{b}.t^{3{\cdot}20}$. The correspondence

* These data on U.S. amosite factory workers, which are censored at age 80, or at the start of other asbestos-related exposure, were not published in this format (Seidman *et al.*, 1979).

between observed and expected numbers is in each case satisfactory beyond 15 years after first exposure, though below 15 years the death rate may be lower than this relationship would predict.

The similarity of the incidence patterns of pleural and peritoneal tumours in U.S. insulation workers noted above was also found in the other studies in which both occurred (shown in Table III) the ratio of pleural to peritoneal remaining about constant in successive periods since first exposure. The comparison of the effects of different forms and intensities of exposure is therefore greatly simplified, as mesothelioma death rates appear to rise approximately as (time since first exposure)$^{3\cdot20}$ irrespective of age, site, fibre type or dust level. This does not, of course, mean that the risk is unrelated to fibre type and intensity of exposure, but that these factors influence only $b$, the constant factor in the incidence formula, $b.t^k$. The incidence at a particular site in any particular cohort can therefore be summarized by the constant $b$. To give some idea of the meaning of this constant, if $b = 4\cdot37 \times 10^{-8}$, the value for insulation workers first employed between 1922 and 1946, the annual death rate 30 years after first exposure will be 2·3 per 1000, and the risk of dying of mesothelioma before age 80 would be $\sim 15\%$ in men first exposed at age 20, allowing for increases in other asbestos-related death-rates.* The risk to older recruits would be very much lower, however. Exposure under similar conditions from age 50, for example, would produce a life-long risk of less than 1%.

We have used 3·20, the estimated exponent of "time since first exposure" based on the mortality data of insulation workers, in the analyses of other studies shown in Table III to simplify the presentation, but any value between 2·5 and 4 would provide an adequate fit to all these cohorts beyond 15 years after first exposure, and would not greatly alter predictions of future rates. In the preceding example, where the observed mesothelioma death rate 30 years after first exposure was $2\cdot3 \times 10^{-3}$ p.a., an exponent of 4 rather than 3·20 would inflate the corresponding predicted lifelong risk to men first exposed at age 20 from 15% to 19%, while an exponent of 2·5 would reduce it to 12%. The standard error of our estimate of the exponent is 0·36, and we would prefer to avoid the spurious precision implied by the second decimal place, which would change if a different estimation procedure were used. (We minimized the $\chi^2$ based on observed and expected cases; the maximum likelihood estimate is 3·17.) Observed mesothelioma rates beyond 40 years after first exposure may be rather too low, due to underdiagnosis in old age, and expected numbers less than 15 years after first exposure based on an exponent of 3·20 appear to be consistently too high (Table III). The true death rate may therefore rise slightly more steeply than our fitted model would suggest, and we prefer the generalization that:

Mesothelioma death rate $\propto$ (time since first exposure)$^{3\cdot5}$, where 3·5 should be interpreted as "between 3 and 4".

### *Relative and absolute incidence of pleural and peritoneal tumours in relation to fibre type*

The values of $b$ (which are measures of relative incidence) for pleural and peritoneal mesotheliomas in the studies shown in Table III are summarized in Table IV. There is an extraordinary difference between different cohorts in the incidence of peritoneal tumours, which constituted between 49% (22/45) of mesotheliomas among factory workers substantially exposed to amosite and other fibres (Newhouse & Berry, 1976), 63% (149/236) in insulation workers, 67% (6/9) in crocidolite gas-mask assemblers in Canada (McDonald & Liddell, 1979), and none among chrysotile (McDonald & Liddell, 1979) or crocidolite miners (Hobbs *et al.*,

* Actuarial calculation, assuming 1977 U.S. white male rates for all causes of death other than mesothelioma inflated by a factor of 1·26, the observed relative risk among insulation workers (Selikoff *et al.*, 1979).

TABLE IV.—*Mesothelioma death rates in various studies, and corresponding predictions of risk. The absolute death rate at a given time since first exposure is proportional to* b, *which is estimated by fitting: death rate* = b (*years since first exposure*)$^{3\cdot20}$ *p.a. The calculation of "lifelong risk", the percentage of similarly exposed men who would die of mesothelioma before age* 80, *is explained in the text*

| Study | Pleural | | Peritoneal | | Total | | Corresponding lifelong risk | | |
|---|---|---|---|---|---|---|---|---|---|
| | Relative risk ($b \times 10^8$) | No. of deaths | Relative risk ($b \times 10^8$) | No. of deaths | Relative risk ($b \times 10^8$) | No. of deaths | Age at first exposure 20 | 30 | 40 |
| Selikoff *et al.* (1979) N. American insulation workers | 1·58 | 65 | 2·79 | 115 | 4·37 | 180 | 15% | 7% | 3% |
| Newhouse & Berry (1976) Factory workers | 2·53 | 23 | 2·42 | 22 | 4·95 | 45 | 17% | 8% | 3% |
| Peto, J. (1980) Chrysotile textile factory | 2·94 | 7 | 0·00 | 0 | 2·94 | 7 | 10% | 5% | 2% |
| Hobbs *et al.*, (1980) Australian crocidolite miners | 5·15 | 26 | 0·00 | 0 | 5·15 | 26 | 17% | 8% | 3% |
| Seidman *et al.* (1979) U.S. amosite factory | 2·45 | 7 | 2·45 | 7 | 4·91 | 14 | 17% | 8% | 3% |

1980) or in factory workers exposed principally to chrysotile (Peto, J., 1980). It thus appears that amphiboles are largely responsible for asbestos-related peritoneal mesotheliomas, although, as none of the 26 mesotheliomas among Australian crocidolite miners (Hobbs *et al.*, 1980) were peritoneal in origin, other factors must also be important. Variation between these cohorts in the incidence of pleural mesothelioma was less, though still substantial. Rates among U.S. amosite workers, English factory workers believed to have suffered substantial exposure to amosite, crocidolite and chrysotile, and English factory workers exposed principally to chrysotile, were similar, but the death rate among U.S. insulation workers was about half that observed in these factories, while that among Australian crocidolite miners was twice that of factory workers, and more then 3 times that of insulation workers.

The corresponding lifelong risks of dying of mesothelioma are also shown in Table IV. The predicted risks are very high for men first exposed at age 20, but very much lower for those exposed at age 30 or later. These figures accord with the observation that more than 10% of deaths occurring 30 or more years after first exposure among North American insulation workers were due to mesothelioma, and a similar projection based on the cohort reported by Newhouse & Berry (1976). (These "lifelong risks" are adjusted for other causes of death, as described in the footnote on page 130. A "lifelong risk" of 7% means that about 7 mesotheliomas will occur by age 80 in a cohort of 100 men followed to extinction. The "cumulative risks" in Fig. 1 are independent of other causes of death; a "cumulative risk" of 7% by age 80 means that 7 mesotheliomas will occur in 100 men who would otherwise have survived to age 80.)

### DISCUSSION

### *Implications for models of carcinogenesis*

The role of ageing in human carcinogenesis is difficult to study directly, and mesothelioma incidence among asbestos workers provides an unusual opportunity to distinguish the influence of age from that of time since first exposure to a carcinogen. For most cancers following ionizing irradiation (Doll, 1978), and for nasal-sinus cancer in early nickel-refining workers (Doll *et al.*, 1970), the absolute risk at a given time after first exposure increases with age at exposure.

Such observations are consistent either with a "multi-stage" model of carcinogenesis, according to which the carcinogen acts at a late stage on a growing number of partially transformed cells, or with the hypothesis that susceptibility to cancer increases with age due to systemic changes. An example of a human cancer for which incidence is clearly unaffected by ageing is therefore useful, and the approximate power-law relationship between mesothelioma mortality and time since first exposure to asbestos also supports Doll's interpretation of other cancer incidence patterns (Doll, 1971; Peto, R., 1977). The fact that certain human and animal tumour rates which rise as the third or higher power of time since exposure are independent of age shows that processes other than age-related susceptibility can and do produce the incidence pattern characteristic of many epithelial cancers. The possibility remains, however, that immune or other regulatory factors are also important for certain tumours, and such theories should be regarded as complementary rather than alternative to the multi-stage model.

Mesothelioma death rates appear to rise as the 3rd or 4th power of time since first exposure in cohorts ranging from amosite workers whose exposures were quite brief to insulation workers exposed for several decades. This would, however, be expected if, as the independence of age suggests, the first step in mesothelial carcinogenesis were affected by asbestos (Day & Brown, 1980). For example, if the contribution to subsequent mortality due to each inhaled fibre were proportional to the cube of time since inhalation, the death rate following brief exposure would rise as the cube of time since first exposure, the rate due to continuous exposure would rise as the 4th power of time, and the effect of intermediate duration would be well approximated by an exponent of time between 3 and 4. Available data do not provide sufficiently precise estimates of the exponent in different cohorts for differences within this range to be detected.

The time dependence (determined by $k$, the exponent in the incidence formula $b.t^k$) may not depend strongly on duration of exposure, but the absolute risk (determined by the constant factor b) certainly does (Newhouse & Berry, 1976; Hobbs *et al.*, 1980). After exposures for up to 10 years, the risk is likely to be roughly proportional to duration, though the effects of longer exposure cannot be predicted without further rather specific assumptions (Peto, J., 1978; 1979).

Incidence and mortality rates are similar for mesothelioma, as the interval from diagnosis to death is usually short. The time taken for the tumour to grow to a clinically detectable size may however be substantial, which could account for the anomalously low mortality 10–15 years after first exposure (Table III). The model $b.(t-10)^2$, a quadratic time-dependence with a lag of 10 years, fits the cohorts shown in Table III better than the model $b.t^{3 \cdot 2}$ up to 15 years after first exposure, and equally well beyond 15 years. Inferences concerning mechanisms of mesothelioma induction should perhaps be based on an exponent of time of about 2 rather than 3 or 4.

In contrast, the age and time dependence of bronchial carcinoma among asbestos workers appear to be quite unlike that of mesothelioma. For bronchial carcinoma the excess risk rises sharply within about 10 years of intense asbestos exposure in middle age (Seidman *et al.*, 1979) and the relative and absolute risks in old age, when most cases occur, appear to be similar irrespective of age at first exposure to asbestos, in striking contrast to mesothelioma (Fig. 1). The ratio of excess lung cancer to mesothelioma thus depends strongly on age at first exposure (Peto, J., 1979).

This is illustrated by the mortality experience up to age 80 of North American insulation workers. Among cigarette smokers aged under 25 at first exposure to asbestos, there were 99 mesotheliomas, and the observed/expected figures for lung cancer were 211/32·67 (relative risk 6·5).

The corresponding respective figures for cigarette smokers aged 25 or over at first exposure were 48 and 237/48·05 (4·9), and for all non-smokers combined 18 and 5/1·04 (4·8). (These expected numbers are calculated from age- and smoking-specific lung-cancer death rates (Hammond *et al.*, 1979). The expected number 1·04 is calculated from lung-cancer rates among non-smoking "blue collar" workers in the American Cancer Society prospective study, while the expected numbers 32·67 and 48·05 are based on rates among smokers.) The ratio of asbestos-induced lung cancer (observed minus expected) to mesothelioma was thus 3·9 among smokers first exposed to asbestos at age 25 or over, 1·8 among smokers first exposed below age 25, and 0·2 among non-smokers. If the ratio of excess lung cancer to mesothelioma continues to fall in this way with reduction in age at first exposure, mesothelioma may constitute the major asbestos-related cancer hazard even among smokers when asbestos exposure begins in childhood. These data also illustrate the approximately multiplicative effects of asbestos and smoking for lung cancer. Mesothelioma incidence is unrelated to smoking, however (Hammond *et al.*, 1979) and among non-smokers the mesothelioma risk is likely to exceed the increase in lung cancer risk irrespective of age at first exposure to asbestos. The ratio of mesothelioma to excess lung cancer could alter at much lower asbestos dust levels, but in the absence of direct evidence of this it seems reasonable to assume that the effect is simply proportional to dust level for both diseases, and that their ratio will depend on age at first exposure in the same way for non-occupational exposure.

The suggestion that the excess relative risk for lung cancer (RR – 1) may be roughly proportional to cumulative asbestos dose (Peto, J., 1978) is now widely accepted as a useful approximation for practical purposes (Acheson & Gardner, 1979). If exactly true, this would suggest that asbestos acts immediately and cumulatively to increase the rate of the final stage in bronchial carcinogenesis, which seems biologically implausible but not impossible. The epidemiological predictions of this model are (1) an abrupt increase in RR after brief intense exposure; (2) a progressive increase during continuous exposure; (3) a constant RR after stopping exposure; (4) an RR (though not absolute risk) at a given cumulative dose independent of both age and age at first exposure; and (5) similar (smoking-specific) RRs in smokers and non-smokers, as smoking seems to have little influence on the final stage (Doll, 1978). These effects could be substantially modified by various observational problems, however, such as the time taken for a tumour to become clinically detectable, and the calculation of accurate smoking-specific expected numbers on which to base RR estimates. For example, the RR for lung cancer among insulation workers rose up to 30 or 35 years after first exposure but then fell (Selikoff *et al.*, 1979), but this decline could be due to cohort changes in exposure, the elimination of heavier smokers and those most heavily exposed to asbestos, and perhaps some reduction in smoking among men with early symptoms of asbestosis. The qualitative conclusion that asbestos acts chiefly at an early stage in mesothelioma induction but affects a later stage or stages for lung cancer seems reasonably secure, but it is difficult to draw any more specific inference.

Other agents also appear to be capable of acting at more than one stage in carcinogenesis, though no single explanation seems likely to encompass the variety of situations in which this occurs. Cigarette smoking probably affects both an early and a late stage in causing lung cancer, and in mice 2-acetyl-aminofluorene seems to act at different stages in different organs (Day & Brown, 1980). Modes of action may also differ between species. Berry & Wagner (1976) have shown that the incidence of mesothelioma in Wistar rats following intrapleural injection of asbestos is significantly higher in animals injected at age 10 months than in those aged 2

months. By a curious quirk of nature, the cancer that has provided the first clear demonstration of human carcinogenesis in which age *per se* exerts little or no influence has thus also provided an example of animal carcinogenesis in which the effect of age is marked.

"Promotion" is used loosely to denote any form of late-stage carcinogenesis, but it is unlikely that all "promoters" act at the same stage or in the same way. Lung-cancer rates in continuing and ex-smokers indicate that cigarette smoke probably acts principally on the first and penultimate stages in lung carcinogenesis (Doll, 1978) but the effect of asbestos, particularly after stopping exposure, is complicated by its persistence in the lung, and epidemiological data alone are unlikely to provide clear evidence on its role as a late-stage carcinogen. Animal experiments in which separate stages of promotion are being investigated directly are now being conducted, however (Slaga *et al.*, 1980) and it seems reasonable to hope that the term "promotion" will eventually be replaced by quantified estimates of effects on specified stages.

### *Extrapolation and industrial hygiene standards*

The suggestion that mesothelioma death rates will rise indefinitely as the 3rd or 4th power of time since first exposure, irrespective of duration of exposure or fibre type, may not be established sufficiently securely to support any particular model of carcinogenesis but, in view of the consistency of the pattern observed in these cohorts, it would be difficult to justify any very different model for the practical purpose of predicting the likely hazards of asbestos exposure in industrial workers (Peto, J., 1978) or future trends in national mesothelioma rates (Peto, J. *et al.*, 1981). The similarity of the absolute incidence rates in these cohorts following such diverse exposures must be largely coincidental and, in the absence of detailed data on duration and level of exposure, any comparison of the effects of different fibre types must be almost meaningless. For example, the risk among Australian crocidolite miners varies in approximate proportion to duration between those employed for less than 3 months and those employed for more than a year (Hobbs *et al.*, 1980) and selection of a suitable subgroup of this cohort could apparently indicate either that crocidolite mining carries a very much higher risk than insulation work, or the opposite.

Long fine fibres are particularly liable to cause mesothelioma in rats, and animal experiments indicate that fibre size and shape are the major determinants of mesothelioma risk following pleural implantation. The risk from glass fibre may thus be similar to that from asbestos fibre of similar dimensions (Bertrand & Pezerat, 1980; Davis *et al.*, 1978; Stanton *et al.*, 1977). It is not known to what extent migration and persistence of carcinogenic activity are also determined by size and shape. Chemical differences between different fibre types may also be important, but until carcinogenic effects of such differences have been demonstrated it would seem sensible to concentrate on fibre dimension rather than mineral type in developing dose-response relationships. For example, airborne chrysotile fibres in a factory environment may be considerably finer than those in a chrysotile mine, and the incidence of mesothelioma at a given nominal fibre count appears to be anomalously low among miners (Acheson & Gardner, 1979). The observation that peritoneal mesothelioma is common among amosite workers and rare or absent among chrysotile workers may also reflect physical differences. The rigidity of amphiboles may be a necessary prerequisite for migration to the peritoneum following inhalation or, perhaps, ingestion, and the difference in the peritoneal: pleural mesothelioma ratio between Australian crocidolite miners (0:26) and crocidolite gas-mask workers in Canada (6:3) probably reflects differences in fibre dimension rather than chemical structure. It may therefore be dangerously optimistic to attribute the substantial incidence of pleural mesothelioma among chrysotile factory workers to occasional crocidolite exposure, merely because mesothelioma is rare among chrysotile miners (Acheson & Gardner, 1979). The overall excess of lung cancer is also relatively low among chrysotile miners, and the only safe conclusion must be that dose-response relationships cannot be expected to apply outside the environment in which they are established, at least until the range of fibre sizes to be included in the fibre count has been chosen less arbitrarily. A single universal standard is liable to be too stringent for certain working conditions and dangerously high for others.

### REFERENCES

Acheson, E. D. & Gardner, M. J. (1979) The ill effects of asbestos on health. In *Asbestos. Vol. 2: Final Report of the Advisory Committee on Asbestos.* London: HMSO.

Berry, G. & Wagner, J. C. (1976) Effect of age at inoculation of asbestos on occurrence of mesotheliomas in rats. *Int. J. Cancer*, **17**, 477.

Bertrand, R. & Pezerat, H. (1980) Fibrous glass: Carcinogenicity and dimensional characteristics. In *Biological Effects of Mineral Fibres, I.A.R.C. Sci. Publ.*, **30**, 901.

Burnet, F. M. (1965) Somatic mutation and chronic disease. *Br. Med. J.*, **1**, 338.

Davis, J. M. G., Beckett, S. T., Bolton, R. E., Collings, P. & Middleton, A. P. (1978) Mass and number of fibres in the pathogenesis of asbestos-related lung disease in rats. *Br. J. Cancer*, **37**, 673.

Day, N. E. & Brown, C. C. (1980) Multistage models and primary prevention of cancer. *J. Natl Cancer Inst.*, **64**, 977.

Doll, R., Morgan, L. G. & Speizer, F. E. (1970) Cancers of the lung and nasal sinuses in nickel workers. *Br. J. Cancer*, **24**, 623.

Doll, R. (1971) The age distribution of cancer: Implications for models of carcinogenesis. *J. R. Statist. Soc. A*, **134**, 133.

Doll, R. (1978) An epidemiological perspective of the biology of cancer. *Cancer Res.*, **38**, 3573.

Hammond, E. C., Selikoff, I. J. & Seidman, H. (1979) Asbestos exposure, cigarette smoking and death rates. *Ann. N.Y. Acad. Sci.*, **330**, 473.

Hobbs, M. S. T., Woodward, S., Murphy, B., Musk, A. W. & Elder, J. E. (1980) The incidence of pneumoconiosis, mesothelioma and other respiratory cancer in men engaged in mining and milling crocidolite in Western Australia. In *Biological Effects of Mineral Fibres, I.A.R.C. Sci. Publ.*, **30**, 615.

Kahn, H. A. (1966) The Dorn study of smoking mortality among U.S. veterans: Report on eight and one half years of observation. *Natl Cancer Inst. Monogr.*, **19**, 1.

McDonald, J. C. & Liddell, F. D. K. (1979) Mortality in Canadian miners and millers exposed to chrysotile. *Ann. N.Y. Acad. Sci.*, **330**, 1.

Newhouse, M. L. & Berry, G. (1976) Predictions of mortality from mesothelial tumours in asbestos factory workers. *Br. J. Indust. Med.*, **33**, 147.

Peto, J. (1978) The hygiene standard for chrysotile asbestos. *Lancet*, i, 484.

Peto, J. (1979) Dose-response relationships for asbestos-related disease: Implications for hygiene standards. II. Mortality. *Ann. N.Y. Acad. Sci.*, **330**, 195.

Peto, J. (1980) The incidence of pleural mesothelioma in chrysotile asbestos textile workers. In *Biological Effects of Mineral Fibres. I.A.R.C. Sci. Publ.*, **30**, 703.

Peto, J., Henderson, B. E. & Pike, M. C. (1981) Trends in mesothelioma incidence in the United States and the forecast epidemic due to asbestos exposure during World War II. In *Quantification of Occupational Cancer.* Eds Schneiderman & Peto. Cold Spring Harbor Laboratory, New York: Banbury Report 9. p. 51.

Peto, R. (1977) Epidemiology, multi-stage models and short-term mutagenicity tests. *Cold Spring Harbor Conf. Cell Proliferation*, **4**, 1403.

Peto, R., Roe, F. J. C., Lee, P. N., Levy, L. & Clack, J. (1975) Cancer and ageing in mice and men. *Br. J. Cancer*, **32**, 411.

Seidman, H., Selikoff, I. J. & Hammond, E. C. (1979) Short-term asbestos work exposure and long-term observation. *Ann. N.Y. Acad. Sci.*, **330**, 61.

Selikoff, I. J., Hammond, E. C. & Seidman, H. (1979). Mortality experience of insulation workers in the U.S. and Canada, 1943–1976. *Ann. N.Y. Acad. Sci.*, **330**, 91.

Slaga, T. J., Fischer, S. M., Nelson, K. & Gleason, G. L. (1980) Studies on the mechanism of skin tumor promotion: Evidence for several stages in promotion. *Proc. Natl Acad. Sci., U.S.A.*, **77**, 3659.

Stanton, M. F., Layard, M., Tegeris, A., Miller, E., May, M. & Kent, E. (1977) Carcinogenicity of fibrous glass: Pleural response in the rat in relation to fibre dimension. *J. Natl Cancer Inst.*, **58**, 587.